# Rapid T_1 _quantification based on 3D phase sensitive inversion recovery

**DOI:** 10.1186/1471-2342-10-19

**Published:** 2010-08-17

**Authors:** Marcel JB Warntjes, Johan Kihlberg, Jan Engvall

**Affiliations:** 1 Center for Medical Imaging Science and Visualization (CMIV), Linköping University, SE58185 Linköping, Sweden; 2 Division of Clinical Physiology, Department of Medicine and Health Sciences, Linköping University Hospital, SE58185 Linköping, Sweden; 3 Division of Radiology, Department of Medicine and Health Sciences, Linköping University Hospital, SE58185 Linköping, Sweden

## Abstract

**Background:**

In Contrast Enhanced Magnetic Resonance Imaging fibrotic myocardium can be distinguished from healthy tissue using the difference in the longitudinal *T*_1 _relaxation after administration of Gadolinium, the so-called Late Gd Enhancement. The purpose of this work was to measure the myocardial absolute *T*_1 _post-Gd from a single breath-hold 3D Phase Sensitivity Inversion Recovery sequence (PSIR). Equations were derived to take the acquisition and saturation effects on the magnetization into account.

**Methods:**

The accuracy of the method was investigated on phantoms and using simulations. The method was applied to a group of patients with suspected myocardial infarction where the absolute difference in relaxation of healthy and fibrotic myocardium was measured at about 15 minutes post-contrast. The evolution of the absolute *R*_1 _relaxation rate (1/*T*_1_) over time after contrast injection was followed for one patient and compared to *T*_1 _mapping using Look-Locker. Based on the *T*_1 _maps synthetic LGE images were reconstructed and compared to the conventional LGE images.

**Results:**

The fitting algorithm is robust against variation in acquisition flip angle, the inversion delay time and cardiac arrhythmia. The observed relaxation rate of the myocardium is 1.2 s^-1^, increasing to 6 - 7 s^-1 ^after contrast injection and decreasing to 2 - 2.5 s^-1 ^for healthy myocardium and to 3.5 - 4 s^-1 ^for fibrotic myocardium. Synthesized images based on the *T*_1 _maps correspond very well to actual LGE images.

**Conclusions:**

The method provides a robust quantification of post-Gd *T*_1 _relaxation for a complete cardiac volume within a single breath-hold.

## Background

Contrast Enhanced Magnetic Resonance Imaging (CEMRI) is the preferred modality for the detection and characterization of myocardial viability [1-6]. At 10-30 minutes after the administration of a *T*_1 _contrast medium fibrotic or otherwise damaged myocardium exhibits hyper-enhancement in comparison with healthy tissue, owing to differences in wash-out kinetics of the contrast agent. Typically, a Phase Sensitive Inversion Recovery (PSIR) sequence is applied for high image contrast between healthy and fibrotic myocardium. In such an acquisition an inversion pulse is applied followed by two acquisitions, one at a short inversion delay time *T*_inv _and a second during the same heart phase at the subsequent heart beat. The total kernel time of the acquisition spans two cardiac RR intervals. The latter acquisition is used to correct the background phase of the former such that a real image is reconstructed instead of a modulus. The advantage of this procedure is that there is no contrast degradation due to signal rectification of the original modulus image [7].

The contrast in the PSIR image is governed by the longitudinal *T*_1 _relaxation of the various tissues. Signal intensity differences in the images indicate differences in *T*_1 _but, since the image is arbitrarily scaled, no absolute numbers can be retrieved. Changes in the absolute relaxation rate *R*_1 _(= 1/*T*_1_) provides a measure for absolute local contrast media concentration [8,9]. Monitoring *R*_1 _over time pictures the actual contrast medium dynamics without the potential offset intensity bias or changes caused by heart rate variability, as seen with conventional dynamic *T*_1_-weigted imaging. Quantification may even improve the stability of segmentation of healthy myocardium and scar tissue. Especially in follow-up studies on the volume of the myocardium and scar, a reliable segmentation is required, independent of scanner settings [10-12]. Finally, *T*_1 _maps are independent of RF coil sensitivity. This may be important for imaging using (phased array) coils with a strong spatial sensitivity gradient and without proper intensity scaling (such as CLEAR, Constant Level Appearing). It may also reduce the need for a fat suppression technique to remove the high-intensity fat signal that may disturb the image reading. A variety of *T*_1 _mapping methods exists (see *e.g*. Refs. [13-17]). A number of these methods rely on strategies with continuous acquisition, which leads to movement artifacts for the heart. Others require several breath-holds to cover the complete cardiac volume. In this work a method is described to retrieve the absolute *T*_1 _relaxation based on a 3D PSIR acquisition, which can cover the complete cardiac volume within a single breath hold.

## Theory

**Figure 1:**
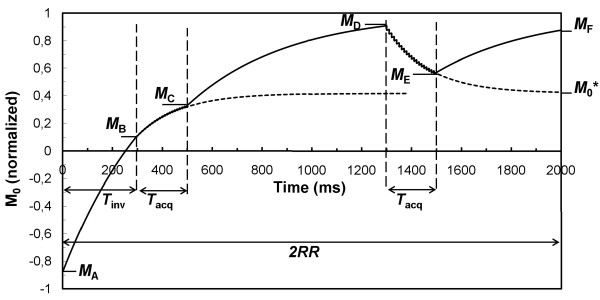
**Schematic representation of the evolution of the magnetization during a 3D PSIR acquisition**. The magnetization evolution is depicted of a tissue with *T*_1 _= 400 ms and an RR interval of 1 s. The steady state magnetization *M*_A_, just after the inversion pulse, relaxes during the inversion delay *T*_inv _towards *M*_B _where the first acquisition starts with a time *T*_acq_. The magnetization is allowed to relax again until the second acquisition at *M*_D_. Finally the magnetization relaxes towards *M*_F_, just before the subsequent inversion pulse. During the acquisition periods the magnetization approaches the saturated magnetization *M*_0_* with an effective relaxation time *T*_1_* (indicated by the dashed lines), depending on the repetition time TR (10 ms) and the applied flip angle α (15°). The acquisition time has a duration of *n *time TR where the TFE factor *n *= 19 in this example.

The evolution of the spin magnetization during a 3D PSIR sequence as a function of time is graphically described in Fig. 1. The fully relaxed magnetization *M*_0 _is normalized to 1. The measurement is repeated every two cardiac RR intervals, where RR is set to 1 s for Fig. 1. To estimate *T*_1 _properly, saturation and acquisition effects on the magnetization must be taken into account. The magnetization starts at a certain steady-state magnetization *M*_A _after the inversion pulse and relaxes with *T*_1 _during the inversion delay time *T*_inv _towards *M*_B_. During the acquisition time *T*_acq _the magnetization evolves under both *T*_1 _relaxation and the continuous application of the RF flip angles α in combination with the subsequent spoiling of the signal, repeated every repetition time TR. This causes a shorter, apparent *T*_1_* relaxation towards a saturated magnetization *M*_0_* as long as the acquisition time *T*_acq _continues [18,19]. The *T*_1_* and *M*_0_* can be found with:(1)

The flip angle α is assumed to be perfect for Eq. 1. It may deviate somewhat due to an imperfect RF slab selection profile and the *B*_1 _inhomogeneity but since the *T*_acq _is short compared to the total RR interval the effect of a deviation on the following equations is small. After the first acquisition the magnetization relaxes again with *T*_1 _from *M*_C _towards *M*_D _where the second acquisition starts. In contrast to the original PSIR measurement identical scanning parameters are applied for the two acquisitions because both are equally important for the *T*_1 _quantification. The magnetization after the second acquisition, *M*_E_, relaxes with *T*_1 _towards the magnetization *M*_F _at 2RR, just before the subsequent inversion pulse. The total magnetization evolution is thus described by:(2)(3)(4)(5)(6)

A perfect inversion pulse is assumed, a small deviation of the complete inversion has only a negligible effect on the calculations. Typically, a 10% reduction of the inversion angle (162 degrees rather than 180) results in an overestimation of 2-3% for a *T*_1 _in the range 200-800 ms. For a single shot PSIR acquisition, with sufficient time between subsequent measurements for the magnetization to fully recover, *M*_A _equals -*M*_0_. For a multi-shot acquisition, either a multi-slice or a 3D sequence, where an inversion pulse is applied every 2RR, in clinical practice a steady-state is reached where *M*_A _= -*M*_F _and (-*M*_A_) <*M*_0_.

Since Eqs. 1-6 are coupled equations there are in fact only 2 unknown parameters, *M*_0 _and *T*_1_, and all 6 magnetizations *M*_A-F _are defined if two of them are known, in case of the 3D PSIR measurement *M*_B _and *M*_D_. A low-high k-space profile order ensures that the image intensity reflects the magnetization in *M*_B _and *M*_D _even though the magnetization changes during the acquisition. The value for *T*_1 _cannot explicitly be calculated and *M*_0 _and *T*_1 _are found using an iterative process. To start the iteration *M*_0 _and -*M*_A _are assumed to be equal to *M*_D_. Using Eq. 2 a coarse *T*_1 _can be calculated according to(7)

Using this *T*_1 _in Eq. 3 *M*_C _is calculated. A new *M*_0 _is then estimated rewriting Eq. 4 as(8)

where exp(*TC) *equals exp(-(*T*_RR _- *T*_acq_)/*T*_1_). Finally *M*_F _is calculated using the new *M*_0 _in Eqs. 5 and 6. The second iteration starts with the estimated *M*_0 _from Eq. 8 and a new *M*_A _= -*M*_F_.

The proposed method is compared to the Look-Locker (LL) sequence [20]. This method can be described as a special case using the same equations. The acquisition is then continuous such that *M*_A _= *M*_B_, *M*_C _= *M*_D _and *M*_E _= *M*_F _and the evolution of the magnetization is in fact described by Eqs. 3 and 5 only. This set of 2 equations can be solved. The steady-state magnetization *M*_t _as a function of time *t *after the inversion pulse is (if the inversion is repeated every RR interval) given by:(9)

Using Eq. 9 the *T*_1_* relaxation can be retrieved from a LL sequence. Subsequently the actual *T*_1 _can be calculated using Eq. 1. From these equations it can directly be seen that the intensity zero-crossing of a LL sequence, in general, does not coincide with the intensity zero-crossing of a PSIR sequence. The continuous acquisition of a LL leads to a *T*_1_* relaxation which is shorter than *T*_1 _and the saturation and acquisition effects lead to a different steady-state magnetization. Therefore care should be taken in using the LL to find the intensity zero-crossing for another sequence, although this is commonly done.

As an additional confirmation of the accuracy of the *T*_1 _quantification the approach of Synthetic MRI [21-24] is applied: The *T*_1 _maps are used as input to simulate a 3D spoiled gradient echo sequence (Inversion Recovery Turbo Field Echo or IR-TFE). Based on the *T*_1 _values of the quantification scan the expected image intensity of an IR-TFE can be calculated for any chosen *T*_inv _using Eqs. 1-6 (using *M*_D _= *M*_E _and a kernel time of a single RR). The synthesized images are compared with the actual ones with the same *T*_inv_. The IR-TFE is interesting since this sequence does not have the second acquisition, like the PSIR method, to restore phase and hence potentially has up to 41% more SNR within the same scan time compared to a PSIR sequence of equal geometry. A prerequisite, however, is that the optimal inversion delay is applied since the IR-TFE scans do suffer from signal rectification in case *T*_inv _is chosen too short [25]. To ensure an optimal *T*_inv _the synthetic images can be set first such that the healthy myocardium appears black. Subsequently this value for *T*_inv _can be used as input for the actual IR-TFE scan.

## Methods

### Phantom measurements

All experiments were performed on a 1.5T Achieva scanner (Philips Healthcare, Best, The Netherlands). Phantoms were made that matched the relevant cardiac relaxation rates as good as possible. Water was used with a 2.5% Agerose solution (Sigma-Aldrich, St. Lious, USA) and different concentrations of the Gadolinium contrast agent (Magnevist, 0.5 mmol/mL, Bayer Healthcare, Germany, diluted to 0.06 - 0.3 mmol/L) resulting in *T*_1 _= 228, 298, 411, 539, 638 and 754 ms. The *T*_2 _relaxation times of all phantoms was in the range 42-59 ms. The 3D PSIR protocol for the *T*_1 _quantification was a segmented 3D Turbo Field Echo Planar Imaging (TFEPI) sequence with an EPI factor 3 and a TFE factor 23. The echo time (TE) was 4.2 ms and the repetition time (TR) 9.4 ms leading to an acquisition phase of 215 ms per heart beat. The matrix size was 228×138 (reconstructed 320×320) over a Field of View (FOV) of 350 × 320 mm. The slices had a thickness of 5 mm (overcontiguous, i.e. the slices overlap). Using a Sense factor 2 a volume of 12-18 slices can be acquired within 24 seconds, depending on the heart rate (2 heart beats per slice). For the phantoms physiology simulation was used for artificial heart triggering. A heart rate of 60 beats per minutes was set, resulting in 12 slices in 24 seconds.

The absolute *T*_1 _relaxation time of the phantoms was validated using a standard inversion recovery sequence with 9 separate measurements at an inversion delay time of 50, 100, 150, 200, 250, 300, 500, 1000 and 2900 ms. The TE was 29 ms (EPI factor 13), TR = 3000 ms and the flip angle 90°.

### In-vivo measurements

For the in-vivo measurements the *T*_inv _was by default set to 300 ms and the flip angle to 18 degrees. The acquisition was performed during diastole. The number of slices was adjusted in the range 12-18 slices to fit within a 24 seconds breath-hold. The quantification method was added to routine clinical examinations of patients that were followed up after primary PCI for ST-elevation myocardial infarction. The study was approved by the regional ethics committee and complied with the declaration of Helsinki. All patients gave written informed consent. They were given 0.2 mmol/kg (max. 15 mmol) Gadolinium contrast agent (Magnevist 0.5 mmol/ml, Bayer Healthcare, Germany). On one patient, the *T*_1 _quantification protocol was performed every 2 minutes. The quantification scan was interleaved with a LL sequence, a single slice inversion recovery scan with a flip angle of 15 degrees and a TR of 25 ms. The LL resulted in 29 heart phases within a breath-hold of 17 seconds. The matrix size was 228 × 201, reconstructed to 320×320.

On all other patients the quantification method was applied once, about 15 minutes after contrast injection. The Synthetic MRI approach was used to establish the optimal inversion delay. Directly after the 3D PSIR acquisition the images were sent to the PACS system (IDS5, Sectra Imtec, Sweden) where the optimal inversion delay time was retrieved from the synthetic images using a dedicated cardiac package (SyMRI Cardiac Studio, SyntheticMR AB, Sweden). The value for the optimal *T*_inv _was used as input for the IR-TFE sequence to ensure black myocardium for this protocol. To compensate for the time delay between the 3D PSIR and the IR-TFE, in general about 1 minute, 20 ms was added to the suggested *T*_inv_.

The IR-TFE sequence was a segmented 3D spoiled gradient echo sequence with TE = 1.3 ms, TR = 4.4 ms and TFE factor 43, leading to an acquisition phase time of 188 ms, also acquired during diastole. In total 17 slices were acquired with a thickness of 5 mm (overcontiguous) and Sense factor 2. The matrix size was 256×172 (reconstructed to 320×320) over a FOV of 350 mm resulting in a scan time of 17 heart beats.

## Results

**Figure 2:**
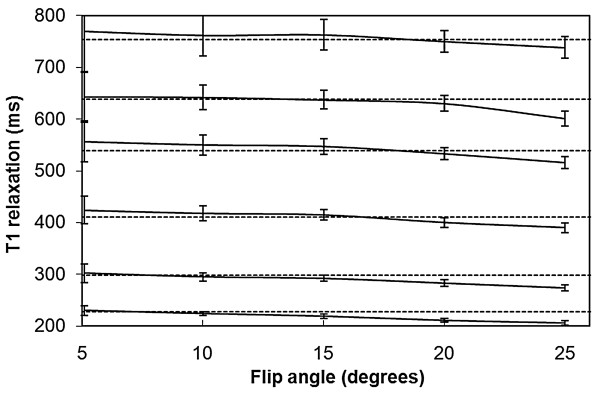
**The measured *T*_1 _relaxation time of various phantoms function of the applied flip angle α**. Six phantoms are shown with different *T*_1 _contrast medium concentration. The inversion delay is 300 ms. For comparison the dashed lines are shown, which represent the *T*_1 _relaxation time as measured with standard inversion recovery. The error bars are the standard deviation of the measurement over 100 pixels.

The measured *T*_1 _relaxation time of various phantoms as a function of the applied acquisition flip angle is shown in Fig. 2 where the *T*_inv _is set to 300 ms. The estimation of the *T*_1 _relaxation is consistent over a large range of applied flip angles. The only exception is the combination of long *T*_1 _and high flip angle when the *T*_1 _time is underestimated. In Fig. 2, the *T*_1 _relaxation time as measured with the standard inversion recovery is displayed as the dotted lines. The standard deviation of *T*_1 _over 100 pixels is shown as the error bars. Based on Fig. 2 the optimal flip angle of the proposed method is in the range 15-20 degrees, with high SNR and good agreement with the standard method.

**Figure 3:**
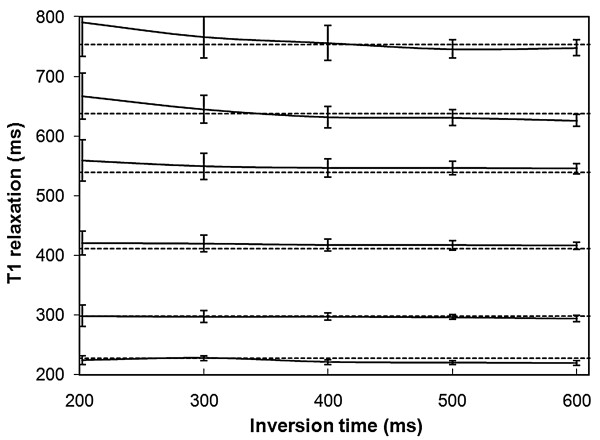
**The measured *T*_1 _relaxation time as a function of the applied inversion delay time**. The same phantoms and settings are used as in Fig. 2. The flip angle was set to 15 degrees.

Fig. 3 exhibits the measured *T*_1 _relaxation time of these phantoms as a function of the applied inversion delay time where the flip angle is set to 15 degrees. A consistent measurement of *T*_1 _is achieved over a large range of applied inversion delays. The combination of very short inversion delay times and high *T*_1 _values may cause an overestimation of the *T*_1 _relaxation time. This is possibly explained in part by the increased Gibbs' ringing at the phantom edges due to the high signal intensity difference. Fig. 3 shows that the inversion delay can be set anywhere in the range of 300 - 600 ms for *T*_1 _values in the range of 200 - 800 ms.

**Figure 4:**
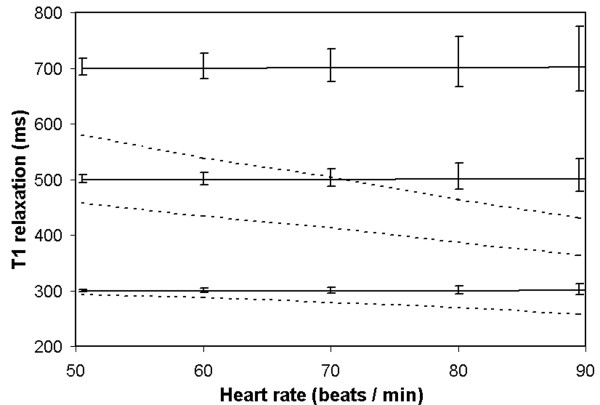
**Simulation of the measured *T*_1 _relaxation times under arrhythmia**. Three phantoms were simulated with *T*_1 _= 300, 500 and 700 ms, respectively. The measured *T*_1 _was plotted as a function of nominal heart rate (solid lines) where heart rate was randomly varied with ± 5% during acquisition. In practical clinical post-Gd cases (HR 60-70, *T*_1 _= 300-500 ms) the potential error due to this arrhythmia, as displayed as the error bars, is 2-6%. Added were the estimated *T*_1 _relaxation times when the acquisition and saturation effects are neglected (dashed lines).

Based on Figs. 2 and 3 the flip angle was set to 18 degrees and the inversion delay time to 300 ms for the post-Gd *in-vivo *measurements. A Monte Carlo simulation was performed with this setting to monitor the potential error of the fitting due to cardiac arrhythmia. The magnetization was calculated using Eqs. 2-6 while the heart rate was varied randomly for each RR-interval in the range ± 5% during the acquisition. In Fig. 4 the estimation of three different *T*_1 _values (300, 500 and 700 ms) at nominal heart rates is displayed. The error bars indicate the standard deviation caused by the random heart rate. The error in *T*_1 _is smaller than ± 4% for 300 ms and smaller than 7% for 500 ms over the entire range of heart rates between 60 and 90 beats per minute. At high *T*_1 _values and high heart rates the error becomes larger, up to 10% for *T*_1 _= 700 ms and a heart rate of 90 bpm. To clarify the importance of taking the actual magnetization behaviour during the sequence into account, the estimated *T*_1 _is shown neglecting the influence of the acquisition and the steady-state saturation effects on the magnetization (Fig 4, dashed lines). The *T*_1 _values are severely underestimated at higher heart-rates. The effects may only be neglected if the RR interval equals more than 4 - 5 times the value of *T*_1_. This occurs only at clinically irrelevant low heart rates.

**Figure 5:**
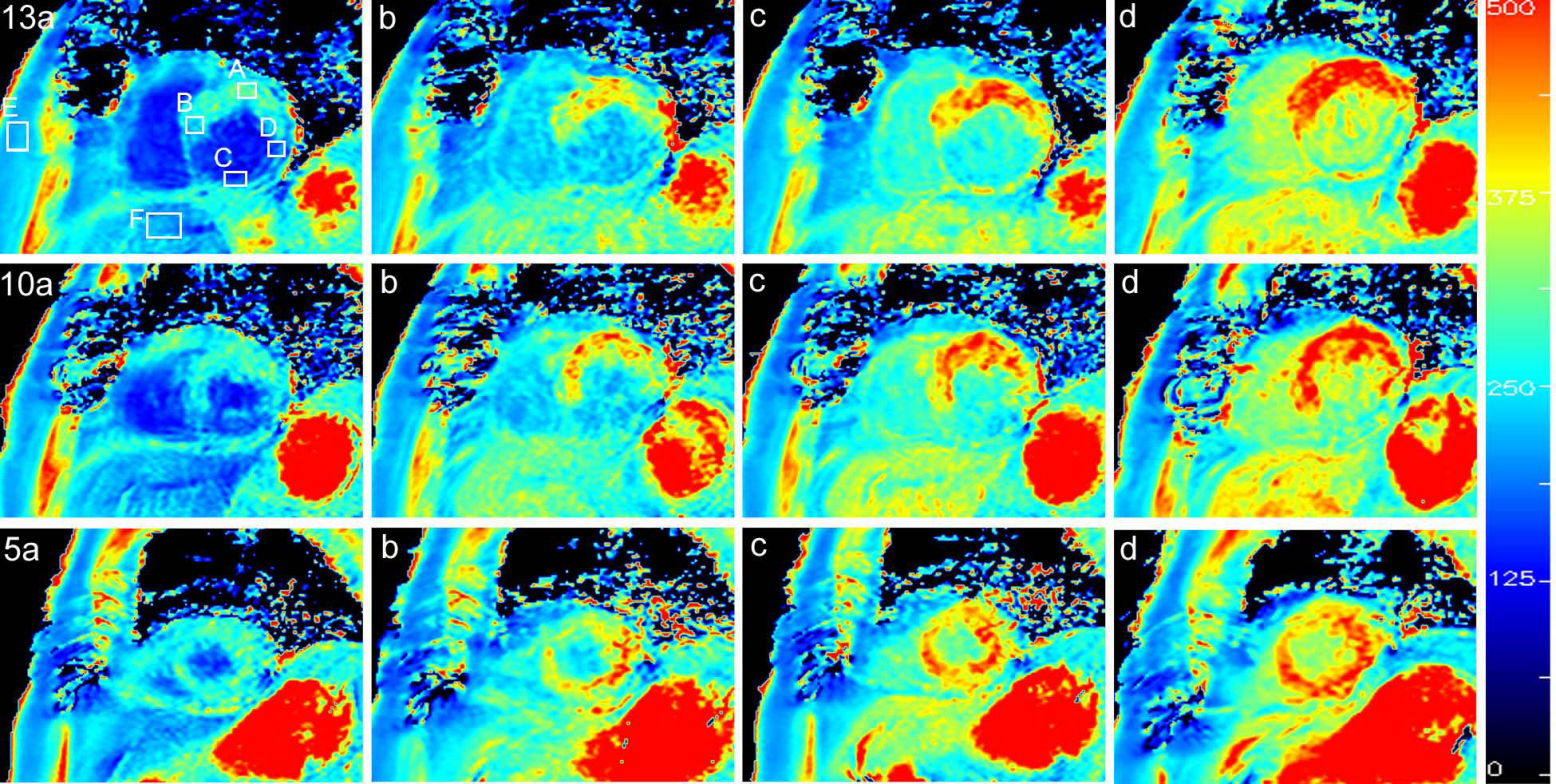
**The absolute *T*_1 _relaxation maps of three short axis slices of the heart**. The slice numbers 13, 10 and 5 out of the 17 that were acquired in a scan time of 19 seconds are shown. The color scale is in the range 0-500 ms. The time after the administration of Gadolinium was **a) **2 min., **b) **6 min., **c) **12 min and **d) **24 min. Indicated in slice 13a are the Regions of Interest that are plotted in the following figures.

**Figure 6:**
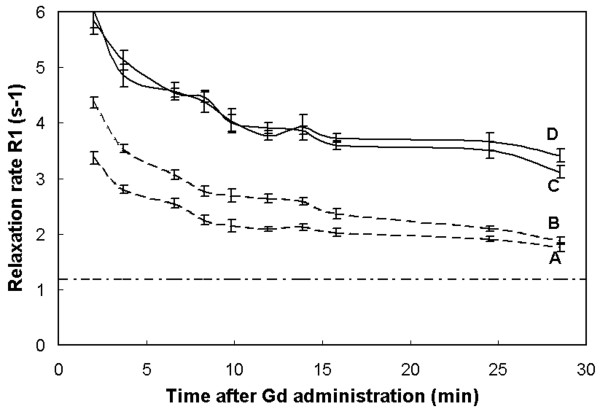
**The absolute relaxation rate *R*_1 _as a function of time for cardiac tissue**. The *R*_1 _as a function of time after Gd administration is shown for the 4 ROIs A, B, C and D, as indicated in slice 13a in Fig. 5. Both A and B are positioned in healthy myocardium, C and D are positioned in fibrotic myocardium. The error bars indicate the standard deviation inside the ROI of ~100 pixels. The dashed-dotted line is the reference *R*_1 _= 1.2 ± 0.2 s^-1 ^of myocardium, obtained before Gd injection.

**Figure 7:**
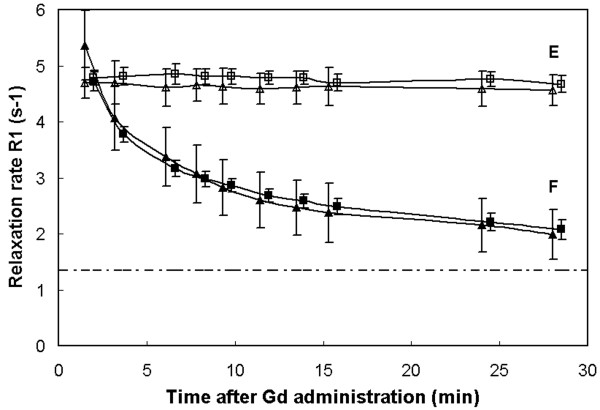
**The absolute relaxation rate *R*_1 _as a function of time for liver tissue and fat**. The *R*_1 _as a function of time after Gd administration is shown for the 2 ROI's E and F, as indicated in slice 13a in Fig. 5. The proposed method (squares) is compared to the Look-Locker method (triangles). ROI E is positioned in subcutaneous fat (open labels), ROI F is positioned on the liver (filled labels). Fat has a stable *R*_1 _= 4.8 ± 0.1 s^-1 ^while the relaxation rate of the liver decreases towards the dashed-dotted line which is the reference *R*_1 _= 1.3 ± 0.2 s^-1^, obtained by our method before Gd injection. The Look-Locker method obtained a reference liver *R*_1 _= 1.3 ± 0.4 s^-1^.

An example of the measured absolute *T*_1 _maps of a short axis slice of a patient with fibrotic myocardium is shown in Fig. 5. The color scale ranges from 0 to 500 ms. Three slices are shown of the 17 that were acquired in the 3D volume (slice number 13, 10 and 5) at various times after the administration of Gd. A clear difference can be seen between the healthy myocardium and the fibrotic myocardium in slices 13 and 10. Six Regions of Interest (ROI) were placed in the images, as displayed in slice 13a, to monitor the relaxation rate *R*_1 _(1/*T*_1_) as a function of time after the Gd injection, where A and B were positioned in healthy myocardium, C and D in fibrotic myocardium, E in the subcutaneous fat and F in the liver. In Fig. 6 the data of the ROIs A-D are shown. The relaxation rate of all myocardial tissue is estimated to be in the order of 6 - 7 s^-1 ^directly after the Gd administration. It rapidly decreases for healthy tissue and at 10-15 minutes post-Gd *R*_1 _is relatively flat at 2 - 2.5 s^-1^. The *R*_1 _of fibrotic tissue, on the other hand, remains high at 3.5 - 4 s^-1^. The dashed-dotted line is the reference *R*_1 _= 1.2 ± 0.2 s^-1 ^for myocardium as measured with our method before the Gd administration. According to Fig. 6 the higher relaxation rate (and hence the hyper-enhancement) of fibrotic tissue compared to healthy tissue is already present after 5 minutes and the difference slowly increases during the following 25 minutes. During this interval the Δ*R*_1 _above the baseline *R*_1 _reduces to about 30% for curves A and B whereas it is only to 50% for curves C and D, indicating faster wash-out kinetics for healthy myocardium. A Look-Locker sequence was applied as well, interleaved between the acquisitions for the proposed new method. This measurement obtained a single slice which was matched geometrically to slice 13 of our method. Since LL is acquired over the complete cardiac cycle direct fitting of *T*_1 _relaxation leads to severe motion artifacts in the region of the heart. The LL measurement is compared with our method for the subcutaneous fat (ROI E in Fig. 5) and for the liver (ROI F), that remain relatively still. As can be seen from Fig. 7, the two methods agree excellently, although the LL results in slightly lower *R*_1 _values, probably due to an imperfect pulse-profile. Note also the difference in standard deviation which for the (single-slice) LL method is 2-3 times that of the proposed method.

**Figure 8:**
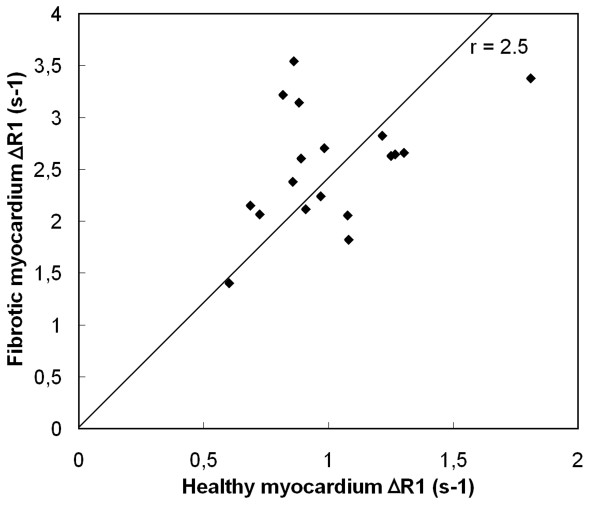
**The change in relaxation rate Δ*R*_1 _after Gadolinium compared to the base line reference *R*_1 _= 1.2 s^-1^, at about 15 post-Gd**. The indicated slope between healthy myocardium and fibrotic myocardium is r = 2.5.

A group of 18 patients was examined about 15 minutes post-Gd when the decrease in relaxation rate is slow. The observed change in relaxation rates Δ*R*_1 _for healthy myocardium and fibrotic myocardium is shown in Fig 8. As base line reference *R*_1 _= 1.2 s^-1 ^is taken. Linear regression estimated a slope between fibrotic and healthy myocardium of r = 2.5 (R^2 ^= 0.93).

**Figure 9:**
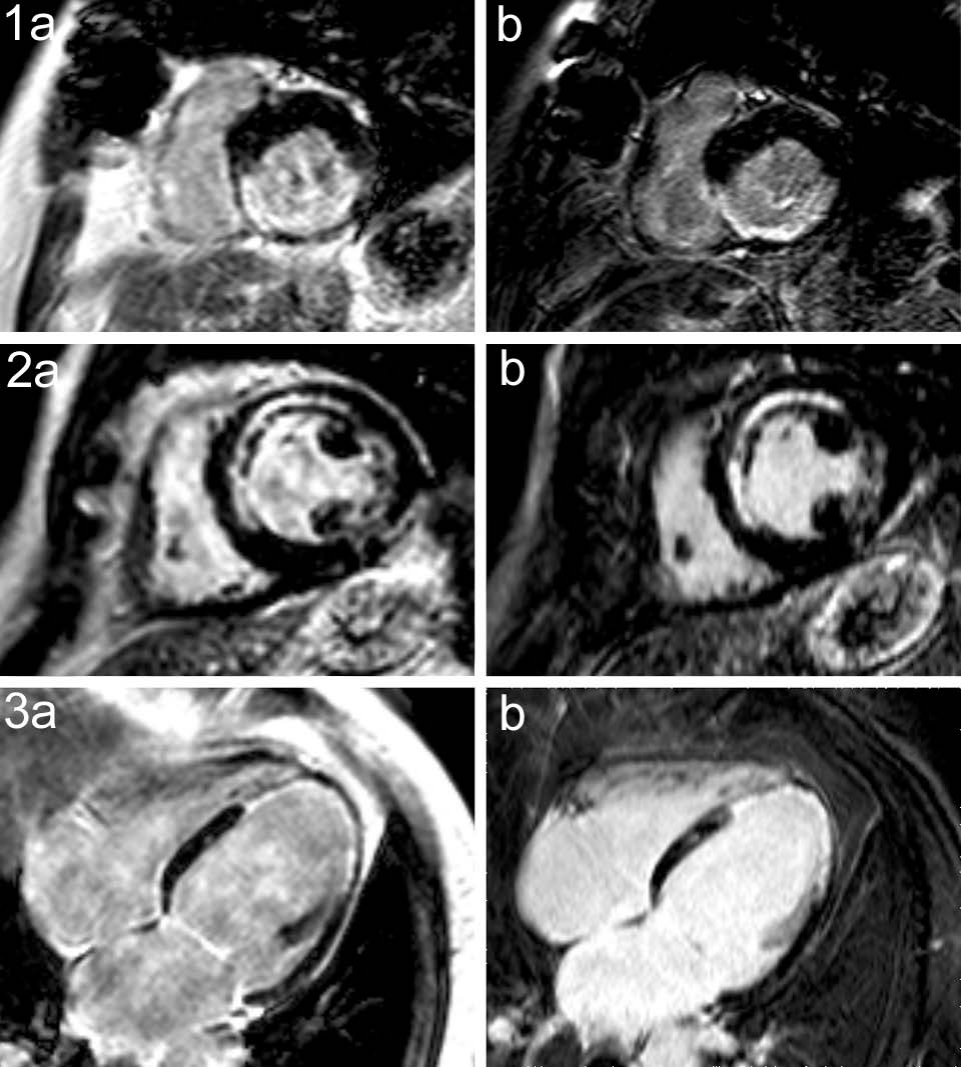
**Comparison of synthesized LGE images with actual LGE images**. Synthesized inversion recovery images (**a**) compared with actual inversion recovery images (**b**) with the same inversion delay plus 20 ms of three different patients. The first patient is the same as in Figs. 5-7 (slice 13). The actual IR-TFE images show pathologies in a similar way but have higher resolution and more slices than the synthesized IR images. The inversion delay was optimal in all three cases based on the prediction of the synthetic images.

As an additional validation the *T*_1 _maps were used as input for synthetic LGE images. Three examples are shown in Fig. 9 of three different patients. The inversion delay was set to a value that turned the healthy myocardium black (1: 234 ms, 2: 272 ms, 3: 267 ms). Next to the synthetic images (a) the corresponding actual IR-TFE measurements are shown (b). The inversion delay of this sequence was set to the predicted value of the synthetic images plus 20 ms to compensate for the time delay between the two scans (about 1 minute in all cases). The first example (1a) corresponds to slice 13, 16 minutes post-Gd as displayed in Fig. 5. Visual assessment shows similar pathologic findings in both image sets. An overall difference in contrast between the synthetic image and the conventional image can be seen due to the lack of a SPIR fat-suppression pulse in the quantification scan.

In order to compare the imaging methods two regions of interest were placed in all images, one in healthy and one in fibrotic myocardium. The Contrast to Noise Ratio (CNR) was defined as the difference in signal intensity of the two ROI's divided by the standard deviation in the ROI over the healthy (black) myocardium. Both methods turn out to have equal CNR (R^2 ^= 0.90). A significant difference is, however, that the IR-TFE acquires more slices (22 - 17 compared to 18 - 10 of the PSIR) and a higher resolution (acquisition voxel size 1.5 × 1.6 mm compared to 1.5 × 2.3 mm of the PSIR) in the same scan time.

## Discussion

The validation of the absolute *T*_1 _relaxation time in phantoms, as depicted in Figs.2 and 3, shows that our method is consistent over a large range of *T*_1 _values, flip angles and inversion delay times. As can be seen in Fig. 2 the optimal flip angle of the method is around 15-20 degrees where the signal to noise ratio is highest and the deviation from the expected value is low. The range of inversion delays that can be chosen is large. The fitting algorithm consistently removes the saturation and acquisition effects. The observation that short *T*_1 _values in the order of 200 ms are consistently measured even with an inversion delay of up to 600 ms implies that the assumption of a perfect inversion pulse is valid. An imperfect inversion would lead to an overestimation of *T*_1_. Possibly this issue has a larger influence at higher field strengths.

Typical *T*_1 _values for an LGE measurement are in the range 200 - 500 ms. Based on Fig 3 the inversion delay of the method can therefore be chosen anywhere in the range 300 - 600 ms. An inversion delay of 300 ms was selected for the *in-vivo *experiments mainly because it is close to the commonly used inversion delay. With these settings the proposed method correctly estimates *T*_1 _values even up to 800 ms although the kernel time is only 2 s. Although it was not the focus of this study it is likely that the method would work for pre-GD myocardial *T*_1 _values as well. In that case a more natural choice for the inversion delay would be higher, e.g. 600 ms.

In clinical practice other parameters are important, such as the variability of the heart-rate during breath-hold, which might decrease the accuracy of the *T*_1 _map. The Monte-Carlo simulation displayed in Fig. 4 shows that small changes in heart rate have less influence on the typical *T*_1 _values than the noise level of the measurement. Furthermore, the resulting error shifts all tissue of interest in a similar fashion and leaves the differences in *T*_1 _between the healthy and fibrotic myocardium virtually unaffected.

Care has to be taken in the interpretation of the absolute *T*_1 _relaxation time. A tissue voxel comprises of many *T*_1 _components rather than the mono-exponential decay that is assumed in the method. Furthermore, at the selected echo time the short-lived *T*_1 _components might be underestimated and the frequency difference between water and fat might lead to a spatial shift of the intensity in the images. Our 2-point method is designed to rapidly estimate the predominant component of *T*_1 _relaxation and for the given scanner parameters this is achieved.

A potential disadvantage of the method is the long breath-hold time (24 s). This results in 12-18 slices of 5 mm depending on the heart rate. Should this be too long the acquisition time may be decreased by reducing the number of slices. The reduced heart volume coverage may be compensated by increasing the slice thickness.

An example of the application of the method is given in Figs. 5, 6, 7 where a patient was monitored every 2 minutes post-Gd. A clear evolution of *R*_1 _is observed over time in the heart and the liver. The change of the relaxation rate Δ*R*_1 _is taken here, rather than *T*_1_, since Δ*R*_1 _is proportional to the absolute amount of contrast medium present in the tissue and should therefore also represent the severity of fibrosis on a microscopic level. As known from practice the Δ*R*_1 _rapidly decreases in the first 5-10 minutes to remain relatively flat in the following 10-30 minutes. The late enhancement contrast already appears after a few minutes. For our patient group the amount of contrast media was about 2.5 times higher in the fibrotic areas compared to the healthy areas (Fig. 8) at about 15 minutes post-Gd. Note that fat is unaffected by the contrast medium and hence can serve as a reference signal intensity for relative measurements of signal intensity during a contrast bolus for perfusion.

The approach of synthetic MRI is used for a direct comparison of the *T*_1 _quantification maps and conventional imaging resulting in very similar images as shown in Fig. 9. Pathology shows up similarly and the method has a good CNR. Interestingly this approach also means that the quantification method may provide both the *T*_1 _maps and the relevant clinical images in one single scan. The ability to synthetically vary *T*_inv _after the actual acquisition may optimize the image quality separately for both ventricles [26]. Moreover the method may serve as a test scan to optimize the *T*_inv _for subsequent IR-TFE sequences.

## Conclusions

We present a method to quantify cardiac *T*_1 _relaxation in a large volume within a single breath-hold, based on a 3D Phase Sensitive Inversion Recovery sequence. The fitting algorithm takes acquisition and saturation effects into account and is robust against variation of scan parameters and heart rate. The method is independent of RF coil sensitivity issues such that high-SNR phased array coils can be employed. The absolute relaxation rate *R*_1 _is monitored over time and over a group of patients. The *T*_1 _maps can be used for 3D segmentation and synthesis of conventional LGE images with a free choice of inversion delay.

## Competing interests

The main author (JBMW) is partly employed by SyntheticMR AB. This software is used additionally for verification of the method.

## Authors' contributions

JBMW developed and verified the theoretical model, JK organized and scanned the patient group and JE was responsible for recruiting, investigating and reporting on the patients. All three reviewed the manuscript.

## Pre-publication history

The pre-publication history for this paper can be accessed here:

http://www.biomedcentral.com/1471-2342/10/19/prepub
